# Suppressing MicroRNA-30b by Estrogen Promotes Osteogenesis in Bone Marrow Mesenchymal Stem Cells

**DOI:** 10.1155/2019/7547506

**Published:** 2019-04-04

**Authors:** Guanqi Liu, Yeming Lu, Zhihui Mai, Runheng Liu, Zhuli Peng, Lin Chen, Zheng Chen, Ruizhi Wang, Hong Ai

**Affiliations:** ^1^Department of Stomatology, The Third Affiliated Hospital of Sun Yat-sen University, Guangzhou, Guangdong, China; ^2^Guanghua School of Stomatology, Hospital of Stomatology, Sun Yat-sen University and Guangdong Provincial Key Laboratory of Stomatology, Guangzhou, Guangdong, China; ^3^Department of Laboratory Medicine, The First Affiliated Hospital, Sun Yat-sen University, Guangzhou, Guangdong, China

## Abstract

MicroRNAs (miRNAs) have been widely demonstrated to interact with multiple cellular signaling pathways and to participate in a wide range of physiological processes. Estradiol-17*β* (E2) is the most potent and prevalent endogenous estrogen that plays a vital role in promoting bone formation and reducing bone resorption. Currently, little is known about the regulation of miRNAs in E2-induced osteogenic differentiation. In the present study, the primary bone marrow mesenchymal stem cells from rats (rBMSCs) were isolated and incubated with E2, followed by miRNA profiling. The microarray showed that 29 miRNAs were differentially expressed in response to E2 stimulation. Further verification by real-time reverse-transcriptase polymerase chain reaction revealed that E2 enhanced the expression of let-7b and miR-25 but suppressed the miR-30b expression. Moreover, a gain-of-function experiment confirmed that miR-30b negatively regulated the E2-induced osteogenic differentiation. These data suggest an important role of miRNAs in osteogenic differentiation.

## 1. Introduction

Osteoporosis is a global public health problem and potentially causes serious fractures, disability, and chronic pain, thus leading to financial burdens for families and lower quality of life for individuals. Estrogen deficiency is one of the main causes of osteoporosis, especially in postmenopausal women [1]. As a steroid hormone, estrogen plays an important role in skeletal homeostasis. Bone remodeling is a process that relies on the dynamic equilibrium between osteoclasts and osteoblasts. It has been well established by both *in vivo* and *in vitro* studies that estrogen inhibits osteoclast formation [2]. Estrogen not only suppresses the formation but also promotes the apoptosis of osteoclasts by regulating the release of cytokines, including interleukin-1, interleukin-6, receptor activator of nuclear factor kappa-B ligand (RANKL), tumor necrosis factor-*α* (TNF*α*), osteoprotegerin (OPG), and macrophage colony-stimulating factor (MCSF) in the bone microenvironment [3–5]. Recently, several studies suggested that estrogen could also inhibit osteoblast apoptosis and promote osteoblast differentiation, thus protecting against bone loss [6, 7]. Estradiol-17*β* (E2) is the most potent estrogen that could effectively improve bone mesenchymal stem cell (BMSC) proliferation and osteogenic differentiation in various species, including mouse, rat, and human [8–10]. However, the precise molecular mechanisms underlying the observed effects of E2 on osteoblast differentiation are still not fully understood. According to its ability to improve bone mineral density and reduce fracture incidence, estrogen replacement therapy is a conventional way to treat osteoporosis. However, studies have shown that the use of estrogen increases the risk of breast cancer, ovarian cancer, thrombotic stroke, and myocardial infarction [11–13]. Therefore, investigating the molecular mechanisms of estrogen-induced osteoblastic differentiation is of great significance because this may potentially inspire precise and targeted therapy for osteoporosis.

MicroRNAs (miRNAs) are a class of small noncoding RNA molecules that govern gene expression at the posttranscriptional level [14]. Expression of miRNA is characteristically spatiotemporal and tissue specific, making them promising targets for precise treatment of various disease [15, 16]. Several miRNAs have been demonstrated to have clear effects on various cancers and are expected to prevent the undesirable effects of conventional treatments. It was suggested that the miRNA replacement therapy in cancers might reduce blood cell reduction, diarrhea, and constipation, all of which could be caused by conventional chemotherapeutic drugs [17]. These implied that it is reasonable to explore the precise osteoporosis treatment from the view of microRNA regulation. Meanwhile, emerging evidence has shown that miRNAs are closely involved in regulating osteogenic differentiation of BMSCs [18]. For example, miR-10a, miR-21, and miR-96 have a positive effect on regulating osteogenic differentiation [19–21], while miR-103a, miR-200a, and miR-141 inhibit osteogenic differentiation [22, 23]. Therefore, it is feasible to analyze the underlying mechanism of estrogen-induced osteogenesis from the view of microRNA regulation and explore the potential precise treatment for osteoporosis based on it.

In this study, we established an *in vitro* model of E2-induced osteogenic differentiation by using rat bone marrow mesenchymal stem cells. Microarray and gain-of-function experiments were performed to analyze the significance of miRNAs in E2-induced osteogenic differentiation. This study may provide the foundation for further work on miRNAs in estrogen-induced osteogenesis and may inspire new strategies to treat bone defects or osteoporosis.

## 2. Materials and Methods

This research was performed under the approval of the Institutional Animal Care and Use Committee of Sun Yat-sen University (Guangzhou, China). All experimental methods were performed in accordance with relevant guidelines and regulations.

### 2.1. Isolation, Culture, and Identification of rBMSCs

Male Sprague-Dawley rats (4 weeks old) were sacrificed by cervical dislocation. rBMSCs were isolated from bilateral femurs and tibias of the rats as described previously [24].

The cells were grown in complete medium (CM), which is Dulbecco's modified Eagle's medium (DMEM, Invitrogen Corp., New York) supplemented with 10% fetal bovine serum (FBS, Gibco, Cat. No. 10099141, New York, USA) and 1% penicillin-streptomycin (Gibco, Cat. No. 15140163) at 37°C in a 5% CO_2_ atmosphere. Cells at passages 3-4 were used in all experiments. To investigate the osteogenic differentiation potential, rBMSCs were exposed to the osteogenic differentiation medium (OM), which is CM supplemented with 10 nM dexamethasone (Sigma-Aldrich, Saint Louis, Missouri, USA Cat. No. D1756), 10 mM *β*-glycerophosphate (Sigma-Aldrich, Cat. No. G9422), and 0.05 mM ascorbic acid-2-phosphate (Sigma-Aldrich, Cat. No. A8960). To investigate the adipogenic differentiation potential, rBMSCs were cultured in adipogenic differentiation medium, which is CM supplemented with 100 nM dexamethasone, 10 ng/ml insulin, and 0.5 *μ*M isobutyl-methylxanthine (IBMX) (Gibco, Cat. No. I7018). For estrogen stimulation, E2 at different concentrations (1 nM, 10 nM, and 100 nM) was added to phenol red-free OM. E2 treatment was continuous during the differentiation.

Immunophenotyping of rBMSCs was analyzed by flow cytometry using fluorescein isothiocyanate-conjugated antibodies which were purchased from eBioscience (California, USA).

### 2.2. Cell Counting Kit-8 (CCK-8) Assay

The toxicity of E2 on BMSCs was measured by the CCK-8 assay. BMSCs were seeded in a 96-well plate and then cultured in CM with different doses of E2 (0 nM, 1 nM, 10 nM, 100 nM, 500 nM, and 1 *μ*M) for 24 h and 48 h. The CCK-8 assays were performed using the CCK-8 Cell Viability Assay Kit (Dojindo, Cat. No. CK04-01, Kumamoto) according to the manufacturers' instruction.

### 2.3. Alizarin Red Staining and Oil Red O Staining

Since the production of calcium nodules needs a long-term stimulation, the rBMSCs were cultured for 5, 9, 13, or 19 days and then subjected to alizarin red staining. rBMSCs cultured in a 6-well plate were washed with PBS and fixed with 70% ice-cold ethanol at 4°C for 1 hour, followed by 1% Alizarin Red (Sigma-Aldrich, Cat. No. A5533) staining for 10 minutes at room temperature. For oil red O staining, rBMSCs cultured in adipogenic differentiation medium for 7 days were washed with PBS and fixed with 4% paraformaldehyde for 15 minutes at room temperature, followed by staining in isopropanol solution of oil red O for 30 minutes at room temperature. Samples were observed under a phase-contrast microscope, and the images were acquired with a scanner. Three independent experiments were performed, and the quantification of the calcium nodules was analyzed by the ImageJ Software based on the whole-well image.

### 2.4. Western Blotting

The expression levels of alkaline phosphatase (ALP), osteocalcin (OCN), osteopontin (OPN), runt-related transcription factor 2 (RUNX2), and bone morphogenetic protein 2 (BMP2) were analyzed by western blotting. The sources of antibodies were as follows: Rabbit monoclonal antibodies against ALP (Cat. No. ab224335), rabbit polyclonal antibodies against OPN (Cat. No. ab104302), mouse antibodies against RUNX2 (Cat. No. ab76956), mouse monoclonal antibodies against BMP2 (Cat. No. ab6285), and mouse polyclonal antibodies against beta-actin (Cat. No. ab8227) were from Abcam, Oxford, UK. Rabbit polyclonal antibodies against OCN were from Bioss Inc. (Cat. No. bs-4917R, Beijing, China). Three independent experiments were performed, and the quantification of the protein band was done by ImageJ software based on both the band area and intensity.

### 2.5. miRNA Microarray

Total RNA of the control cells (cultured in OM) and the E2-incubated rBMSCs (cultured in OM supplemented with 100 nM E2 for 13 days) was isolated with TRIzol (Invitrogen, Cat. No. 15596018, California, USA) and purified with the RNeasy Mini Kit (Qiagen, Cat. No. 74104, Hilden, Germany) according to the manufacturer's instructions. RNA quality and quantity were measured by a NanoDrop spectrophotometer (ND-1000, NanoDrop Technologies Inc.), and RNA integrity was determined by gel electrophoresis. The miRCURY™ Hy3™/Hy5™ Power Labeling Kit (Exiqon, Cat. No. 208035, Denmark) was used for miRNA labeling according to the manufacturer's guideline. After the labeling procedure, the Hy3™-labeled samples were hybridized on the miRCURY™ LNA Array V19.0 (Exiqon) according to the array manual. Then, the slides were scanned by the Axon GenePix 4000B Microarray Scanner (Axon Instruments, USA).

Scanned images were then imported into the GenePix Pro Software V6.0 for grid alignment and data extraction and normalization. A median normalization method was used. Differentially expressed miRNAs between two groups were identified with the fold-change thresholds of >1.5 or <0.67. Finally, a hierarchical clustering was performed to show the distinguishable miRNA expression profiling between these two groups. The heat map of the differentially expressed miRNAs was made by the Mev clustering software based on the normalized intensities of each group [25]. The pathway analysis was performed by the Nimble Scan V2.5 (Roche NimbleGen Inc., Madison, WI, USA).

### 2.6. Quantitative Reverse Transcriptase PCR (qRT-PCR) of miRNA

Total RNAs were isolated with TRIzol and transcribed into cDNA with the Thermo Scientific RevertAid First Strand cDNA Synthesis Kit (Thermo Fisher Scientific, Cat. No. K1622, California, USA) together with bulge-loop miRNA primers (RiboBio, Guangzhou, China) according to the manufacturer's guidelines. RT-PCR was performed with the SYBR Green detection reagent (Tiangen, Cat. No. FP209-01, Beijing, China). All reactions were run in triplicate, and the miRNA levels were normalized to U6 snRNA.

### 2.7. Cell Transfection

miRNA mimics and inhibitors were synthesized and purified by RiboBio. The E2-treated rBMSCs were transfected with 50 nM miRNA mimics or 400 nM miRNA inhibitors. Transfection was performed with Lipofectamine RNAiMAX Reagent (Invitrogen, Cat. No. 13778030) according to the manufacturers' instruction.

### 2.8. Statistical Analysis

SPSS statistical software V20.0 was used for data analysis. The data were presented as the mean ± standard error of the mean. Statistical significance was analyzed with one-way ANOVA. *p* < 0.05 was considered statistically significant.

## 3. Result

### 3.1. Identification of Rat Bone Marrow Mesenchymal Stem Cells (rBMSCs)

On the third passage, the isolated rBMSCs displayed rapid proliferation and a fibroblast-like appearance. A flow cytometry assay revealed that the rBMSCs were positive for mesenchymal marker CD29 and stem cell marker CD90 but were negative for myelogenous markers CD11b/c and CD45 (Figure S1A). Next, the isolated rBMSCs were analyzed for their capacity of multidirectional differentiation, since rBMSCs are capable of differentiating into osteogenic or adipogenic lineages. As expected, rBMSCs that were cultured in the osteogenic differentiation medium (OM) displayed significant calcium deposits, which indicate osteogenic differentiation, compared with those cultured in complete medium (CM) (Figure S1B). Furthermore, rBMSCs that were cultured in adipogenic differentiation medium accumulated lipid droplets, indicating the adipogenic differentiation (Figure S1C).

### 3.2. The Viability of rBMSCs Cultured with E2

**Figure 1:**
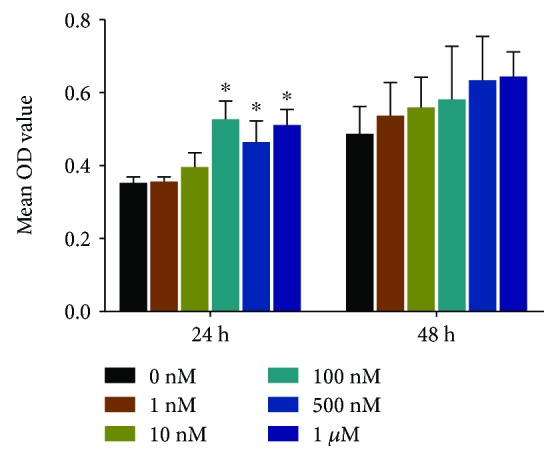
The proliferation of BMSCs stimulated with a range of concentrations of E2 (0 nM, 1 nM, 10 nM, 100 nM, 500 nM, and 1 *μ*M) for 24-48 h. No significant toxic effect was observed among these concentrations (^∗^ *p* < 0.05).

To explore whether E2 would affect the viability of rBMSCs, the cells were treated with a range of concentrations of E2 (0 nM, 1 nM, 10 nM, 100 nM, 500 nM, and 1 *μ*M) for 24-48 h and then CCK-8 assays were performed. No significant differences in toxic effect were observed among these concentrations (Figure 1).

### 3.3. E2 Induced the Osteogenic Differentiation in rBMSCs

**Figure 2:**
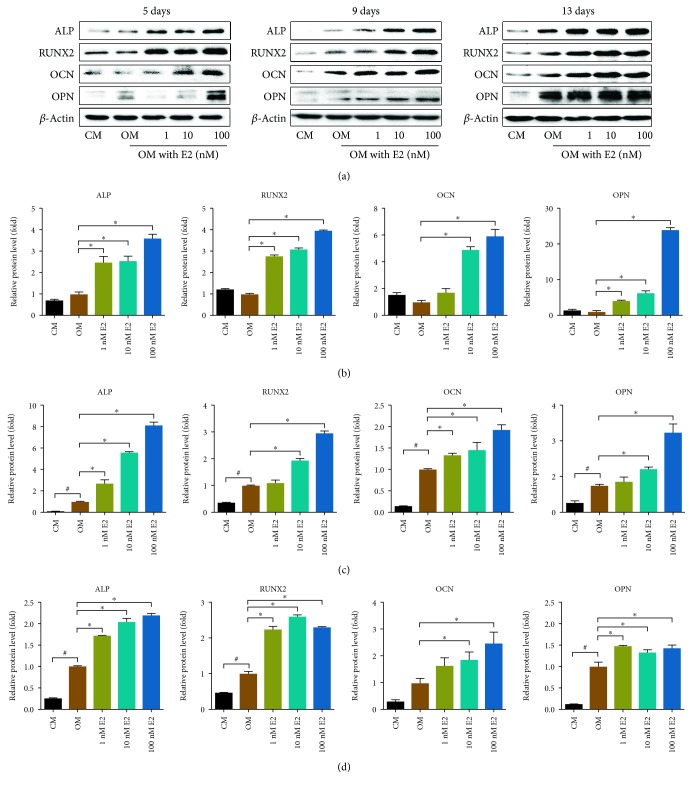
The effect of E2 on the expression of osteogenesis-related proteins. (a) Western blotting showed that the expression of ALP, RUNX2, OCN, and OPN was increased by E2 at various concentrations and duration times in rBMSCs. (b–d) The relative protein levels were calculated based on the loading control for day 5 (b), day 9 (c), and day 13 (d). CM: complete medium; OM: osteogenic medium. Three independent experiments were performed. (^∗^OM group vs. 1-100 nM E2 group, *p* < 0.05; ^#^CM group vs. OM groups, *p* < 0.05.)

To confirm the effect of E2 on osteogenic differentiation, the rBMSCs were treated with different doses (1 nM, 10 nM, and 100 nM) of E2 for 5, 9, or 13 days, followed by immunoblotting analysis for the expression of osteogenesis-related proteins, including ALP, RUNX2, OCN, and OPN. As shown in all three incubation periods, E2 treatment resulted in a significant increase of ALP, RUNX2, OCN, and OPN levels consistent with the series of E2 concentrations (Figure 2).

**Figure 3:**
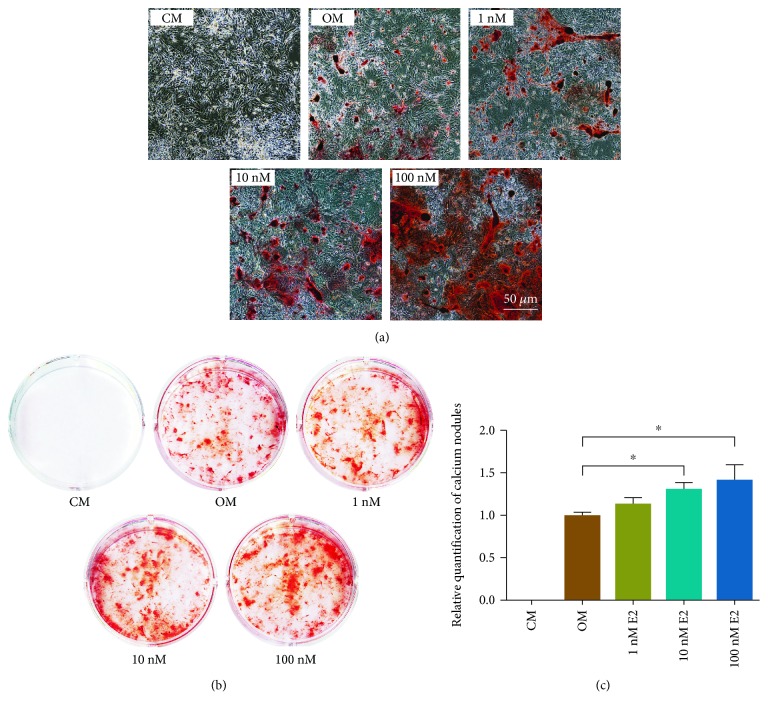
Alizarin red staining showed that E2 promoted the production of calcium nodules in a dose-dependent manner. (a) Calcium nodules observed in a microscope. (b) Whole-well images of calcium nodules. (c) Relative quantification of calcium nodules performed in the whole-well images. CM: complete medium; OM: osteogenic medium. Three independent experiments were performed. (Bar = 50 *μ*m. ^∗^OM group vs. 1-100 nM E2 groups, *p* < 0.05).

The effect of E2 on the production of calcium nodules was further investigated. The alizarin red staining assay revealed that after being cultured in OM for 13 and 19 days, rBMSCs exhibited significant calcium nodule formation, which was not observed in cells cultured in CM. Moreover, E2 addition to OM further promoted the production of calcium nodules in rBMSCs in a dose-dependent manner (Figure 3 and Figure S2). Taken together, the above results indicate that E2 enhanced the osteogenic differentiation of rBMSCs.

**Figure 4:**
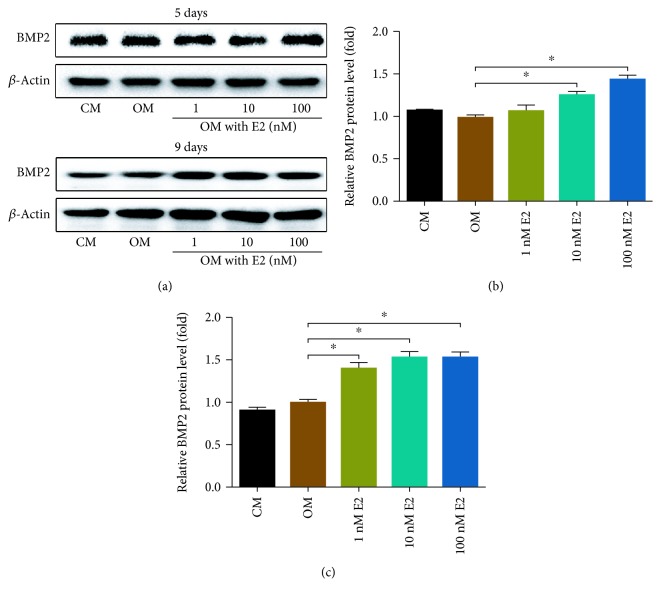
The effect of E2 on BMP2 expression. (a) Western blotting shows that the expression of BMP2 was increased with 10 nM and 100 nM E2 stimulation for 5 days. The expression of BMP2 was also increased with 1 nM, 10 nM, and 100 nM E2 stimulation for 9 days. (b–c) The relative protein levels were calculated based on the loading control for day 5 (b) and day 9 (c). CM: complete medium; OM: osteogenic medium. Three independent experiments were performed. (^∗^OM group vs. 1-100 nM E2 group, *p* < 0.05.)

In order to explore the potential mechanism of E2-induced osteogenic differentiation, the expression of BMP2 was detected in the early stage of E2 stimulation. As shown in Figure 4, the expression of BMP2 was increased with 10 nM and 100 nM E2 stimulation for 5 days. After the stimulation of E2 for 9 days, the expression of BMP2 was also increased in the 1 nM, 10 nM, and 100 nM group. Therefore, BMP2 was involved in the regulation of E2-induced osteogenic differentiation.

### 3.4. E2-Induced Osteogenesis Involved Altered miRNA Expression

**Figure 5:**
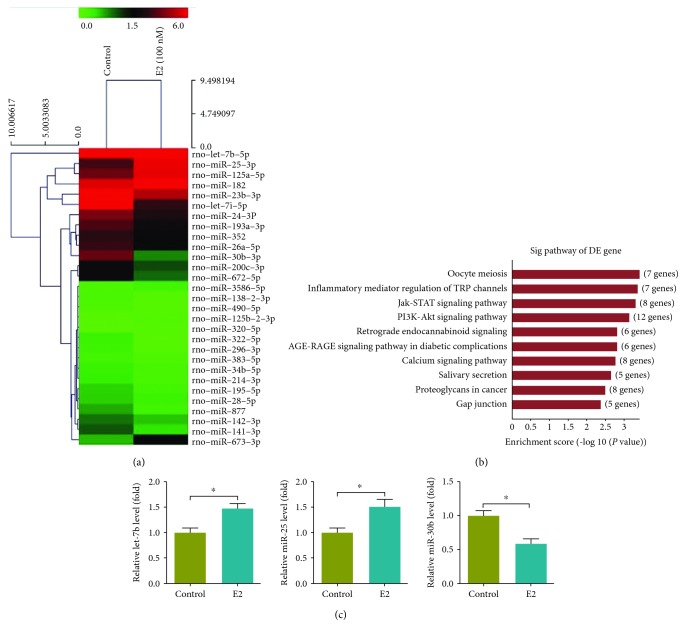
Altered miRNA expression in the process of E2-induced osteogenic differentiation. (a) Heat-map representation of miRNAs differentially expressed in control and E2-treated rBMSCs. Red color indicates the miRNAs that were induced, and green color indicates miRNAs that were repressed. (b) Pathway analysis of the altered miRNAs. (c) E2 significantly increased the expression of let-7b and miR-25, while decreasing the expression of miR-30b. Three independent experiments were performed. (^∗^Control group vs. E2 group, *p* < 0.05.)

To investigate the miRNA expression in E2-induced osteogenic differentiation in rBMSCs, the total RNAs were extracted from E2-treated or nontreated cells for microarray screening. Among the 700 miRNAs represented on the chip, 29 were differentially expressed in response to E2 treatment (Figure 5(a)). Most of these miRNAs (21/29) were downregulated, while 8 of them were upregulated compared with the control group during this time frame (Table 1 and Table S1). Consistently, the majority (19/29) of these miRNAs have been categorized as related to osteogenic differentiation in previous studies (Table S2).

Potential target genes of these 29 miRNAs were predicted using the databases Microcosm, Miranda, and Mirdb. The genes that were identified as targets of the 29 miRNAs by all three databases were further subjected to the pathway enrichment analysis. The KEGG pathway analysis showed that the predicted target genes were enriched in ten signaling pathways (Figure 5(b)). Among these pathways, JAK-STAT signaling, PI3K-AKT signaling, and calcium signaling have been proven to be closely related to osteogenesis or bone metabolism [26–28]. These results implied that miRNAs may be involved in the regulation of E2-induced osteogenesis.

We selected 3 miRNAs (let-7b, miR-25, and miR-30b) to confirm their alteration induced by E2, using a quantitative PCR assay. As shown, the expression levels of let-7b and miR-25 were upregulated 1.5-fold, while the expression level of miR-30b dropped by 58%, compared with the control groups (Figure 5(c)). These results suggest that E2 promoted the expression of let-7b and miR-25 and decreased the miR-30b expression.

### 3.5. miR-30b Regulated the E2-Induced Osteogenesis

**Figure 6:**
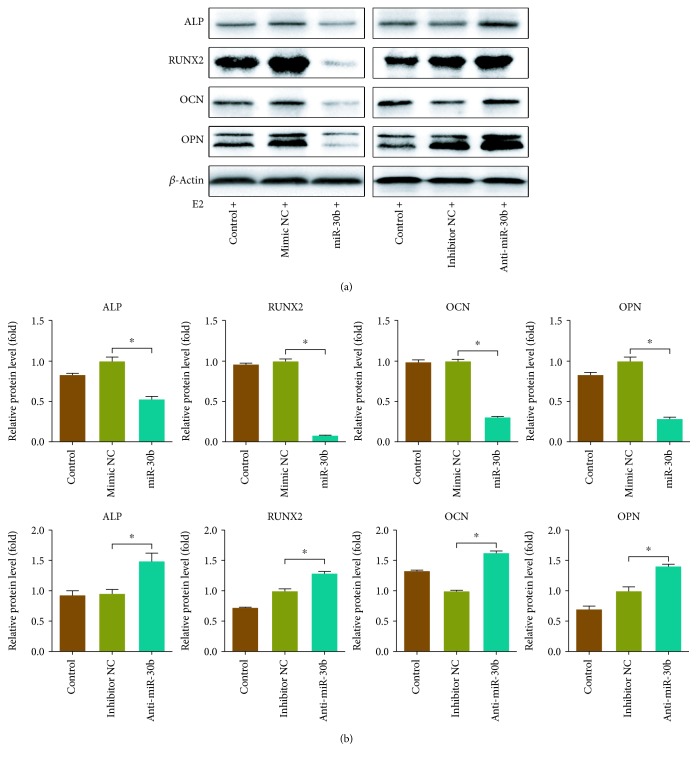
The effect of miR-30b on the expression of osteogenesis-related proteins. (a) Overexpression of miR-30b attenuated the expression of ALP, RUNX2, OCN, and OPN, while suppression of miR-30b increased the expression level of these proteins. (b) The relative protein levels were calculated based on the loading control. Three independent experiments were performed. (^∗^Mimic NC group vs. miR-30b group, or inhibitor NC group vs. anti-miR-30b group, *p* < 0.05.)

**Figure 7:**
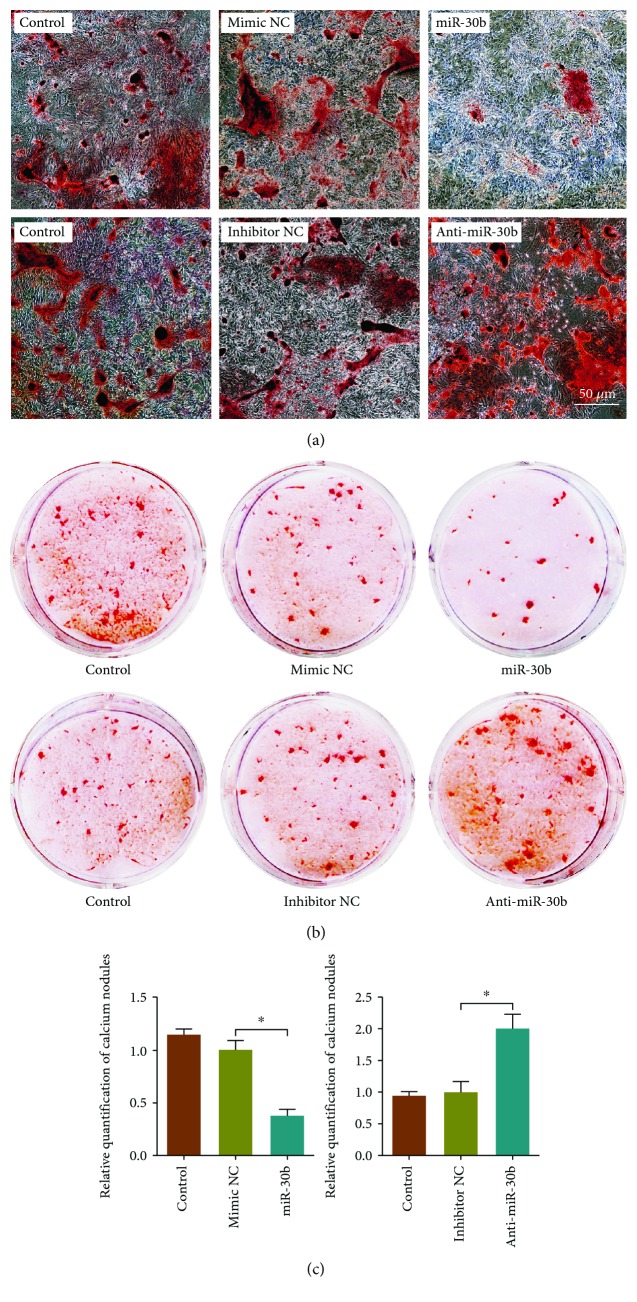
Alizarin red staining shows that overexpression of miR-30b limited the production of mineralized nodules, while the suppression of miR-30b improved the production of mineralized nodules. (a) Calcium nodules observed in a microscope. (b) Whole-well images of calcium nodules. (c) Relative quantification of calcium nodules performed in the whole-well images. Three independent experiments were performed. (Bar = 50 *μ*m. ^∗^Mimic NC group vs. miR-30b group or inhibitor NC group vs. anti-miR-30b group, *p* < 0.05.)

miR-30b was chosen for an investigation of its effect on E2-induced osteogenic differentiation. Immunoblotting assay revealed that overexpression of miR-30b markedly attenuated the expression of E2-induced osteogenesis-related proteins, including ALP, RUNX2, OCN, and OPN, while knockdown of miR-30b significantly increased the expression of these proteins (Figures 6(a) and 6(b)). Furthermore, alizarin red staining revealed that the production of a mineralized nodule in E2-treated rBMSCs was significantly suppressed by miR-30b overexpression, but it was apparently restored by miR-30b silencing (Figure 7). These data indicated that miR-30b may negatively regulate E2-induced osteogenesis.

## 4. Discussion

Hormone replacement therapy (HRT) is a common treatment for osteoporosis. However, its long-term use is restricted by potential complications [29, 30]. Understanding the cellular and molecular mechanisms of the estrogen-induced effects on osteoporosis may provide novel and precise treatments, which could avoid these systemic side effects. miRNAs are considerably small molecules and their expression is strictly spatiotemporal and tissue specific [31], making them promising targets for the precise treatment of various diseases. Our study revealed the miRNA expression profile during estrogen-promoted osteogenic differentiation and explored the role of miR-30b in this process, indicating that miRNA-targeted treatment could be a new strategy for osteoporosis therapy.

According to previous studies, the concentration of E2 is used to induce osteogenic differentiation ranging from 0.1 to 100 nM [32–34]. Moreover, the cytotoxicity test of E2 revealed that the concentration of E2 from 1 nM to 1 *μ*M was nontoxic to BMSCs. Therefore, we choose 1 nM to 100 nM E2 in this study. According to our results, 100 nM E2 has been found to have an obvious osteogenic effect on BMSCs.

In this study, we showed that E2 promoted the expression of ALP, RUNX2, OCN, and OPN. An osteoblast expresses a high level of ALP, which has been widely used as an early-stage marker of osteoblast differentiation [35]. RUNX2 is upregulated during osteogenesis [36] and promotes the differentiation of multipotent mesenchymal cells into osteoblasts and inhibits their differentiation into adipocytes [37]. OCN is closely related to the calcium-ion balance and mineralization in bone tissue [38], and OPN regulates the formation of hydroxyapatite and promotes mineralization of the bone matrix. Both OCN and OPN are related to the late-stage osteoblast differentiation [39]. This may account for our observation of inconspicuous upregulation of OCN and OPN at 1 nM and 10 nM doses of E2 at the early time point (day 5). BMP2 is an important early-stage regulator of osteogenesis [40, 41]; after E2 stimulation, the expression of BMP2 was upregulated significantly in the early stages, indicating that BMP2 was involved in the regulation of E2-promoted osteogenesis.

let-7b, miR-25, and miR-30b were selected to validate the microarray results via a quantitative PCR assay. The reasons for selecting these miRNAs to investigate the effect on E2-induced osteogenic differentiation are as follows: (1) The expression levels of these miRNAs are relatively high in rBMSCs according to the intensity values detected by microarray analysis (Table 1), which facilitates verification by quantitative PCR assay. (2) Among the 29 miRNAs, the alterations in expression of these miRNAs are the most significant. (3) It has been suggested that these miRNAs were closely related to mineralization [23] and osteogenesis [18]. One target gene of miR-30b is RUNX2, which is an important osteogenic differentiation marker and was found to be upregulated by E2 in the present study. Hence, miR-30b was more likely to regulate E2-induced osteogenic differentiation and was therefore selected to investigate its effect. Both the let-7 family and miR-25 have been found to be related to osteogenesis in a previous study. For example, the let-7 family was able to enhance the osteogenesis and repress the adipogenesis of human stromal/mesenchymal stem cells [42], and miR-25 played a role in regulating osteoblast differentiation in the osteoblast-like line MG-63 [43]. However, whether they were involved in the regulation of E2-induced osteogenic differentiation was still unknown. Our study provided a first clue that both let-7b and miR-25 play a positive role in E2-induced osteogenesis in rBMSCs.

**Figure 8:**
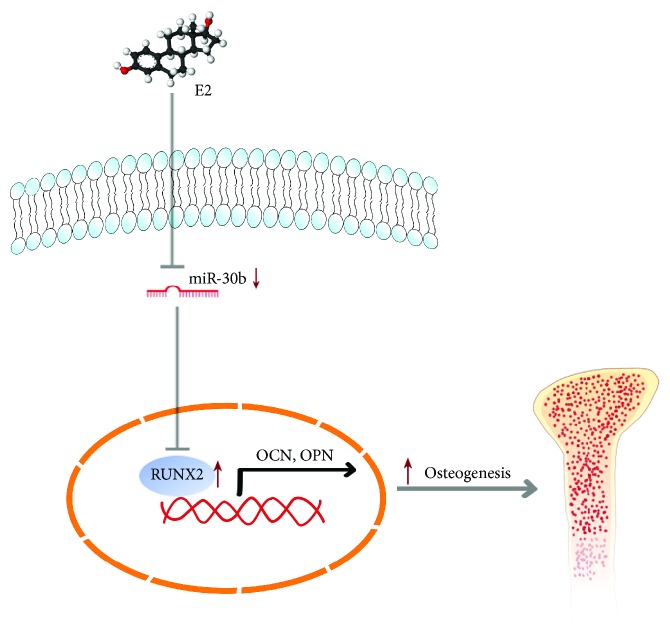
The schematic diagram shows that E2 promotes the osteogenic differentiation of rBMSCs by downregulating the expression of miR-30b.

The expression of miR-30b was decreased in E2-induced osteogenesis. It has been reported that BMP2 promoted vascular smooth muscle cell (VSMC) calcification by downregulating miR-30b and miR-30c expression [44]. The process of vascular calcification is highly similar to physiological mineralization, which consists of the degradation of pyrophosphate by alkaline phosphatase and the deposition of hydroxyapatite crystals on the collagen-rich matrix [45, 46]. Our data further implied that miR-30b negatively regulated E2-induced osteogenesis by interaction with RUNX2 (Figure 8). Meanwhile, Runx2 is one of the downstream factors of the BMP2 pathway. Therefore, E2 is supposed to promote osteogenesis via BMP2/miR-30b/Runx2 signaling. In addition, the expression of ALP, OCN, and OPN was inhibited by miR-30b. ALP, OPN, and OCN were not predicted as the target genes of miR-30b; therefore, the expression of these three proteins might not have been regulated by miR-30b directly but rather by other factors or molecular pathways. As a transcription factor, RUNX2 transactivates the expression of OPN and OCN [47–49]. Therefore, miR-30b may inhibit the expression of OPN and OCN via RUNX2.

On the other hand, miR-30b can inhibit autophagy by directly targeting beclin1 (BECN1) and autophagy protein 5 (ATG5) [50]. Recent studies have found that autophagy promotes osteogenic differentiation of MSCs [6, 51]. Therefore, autophagy may be another possible mechanism underlying the inhibition of osteogenic differentiation by miR-30b. However, this speculation requires more investigation for confirmation.

## 5. Conclusions

In conclusion, our study demonstrates that E2 can effectively promote osteogenic differentiation of rat BMSCs and can provide an insight into the potential contribution of miRNAs to E2-induced osteogenesis. These findings inform us that miR-30b can be a possible therapeutic target to treat osteoporosis. Further *in vivo* experiments are needed to support its application.

## Figures and Tables

**Table 1:** Data of the altered miRNAs. The basal intensity values and fold change (E2/control) of the differentially expressed miRNAs are shown. The fold change = normalized data in the E2 group/normalized data in the control group (shown in Table S1).

miRNA	Basal intensity value		Fold change	miRNA	Basal intensity value		Fold change
	Control	E2			Control	E2	
miR-25	646.50	1271.00	1.83	miR-138-2	47.50	30.00	0.59
let-7b	1494.00	2841.50	1.77	miR-214	85.50	56.50	0.62
miR-125a	781.00	1258.00	1.50	miR-24	826.00	548.00	0.62
miR-182	1147.00	1897.00	1.54	miR-296	69.50	30.50	0.41
miR-320	18.00	30.50	1.58	miR-672	370.00	209.00	0.53
miR-125b	20.00	32.50	1.52	miR-877	142.50	73.00	0.48
miR-3586	43.00	69.50	1.51	miR-383	65.00	41.00	0.59
miR-673	130.50	328.00	2.34	miR-490	38.00	25.50	0.63
miR-30b	771.00	183.00	0.22	miR-352	568.50	396.00	0.65
miR-322	77.50	24.00	0.29	miR-28	116.50	81.50	0.65
miR-26a	607.50	321.00	0.49	miR-142	187.50	134.00	0.67
let-7i	1316.50	628.50	0.45	miR-195	114.00	66.50	0.54
miR-23b	1623.00	1086.50	0.62	miR-34b	82.00	57.50	0.65
miR-193a	676.00	418.50	0.58	miR-200c	369.50	238.00	0.60
miR-141	213.00	107.00	0.47				

## Data Availability

The data used to support the findings of this study are available from the corresponding author upon request.

## Supplementary Materials

Supplementary 1 Table 1: detailed data of the altered miRNAs. The fold change (E2/control), basal intensity values, and the normalized data of the differentially expressed miRNAs are shown. The fold change = normalized data in the E2 group/normalized data in the control group.

Supplementary 2 Table 2: the effect of miRNAs in previous studies. The effect of miRNAs in previous studies were presented. P: positive; N: negative; MSCs: Mesenchymal Stem Cells; BMSCs: Bone Mesenchymal Stem Cells; SMCs: Smooth muscle cells; mADSCs: mouse Adipose-Derived Stem Cells; hADSCs: human Adipose-Derived Stem Cells; hMSCs: human Mesenchymal Stem Cells; PMCO: Primary Mouse Calvaria Osteoblasts; PDLF: Periodontal Ligament Fibroblast.

Supplementary 3 Figure 1: identification of rBMSCs. (a) Flow cytometry assay shows the surface markers of rBMSCs. Isotype controls are presented as red plots, and the specific cell surface markers are presented as blue plots. The isolated rBMSCs were positive for CD29 and CD90, both of which are the markers of rBMSCs, but were negative for myelogenous makers CD11b/c and CD45. (b, c) The multidirectional differentiation abilities of rBMSCs. (b) The alizarin red staining showed that rBMSCs cultured in the osteogenic differentiation medium displayed significant calcium deposits compared with the control group, and (c) the oil red O staining showed that rBMSCs cultured in adipogenic differentiation medium could accumulate lipid droplets compared with the control group. Three independent experiments were performed. (Bar = 100 *μ*m.)

Supplementary 4 Figure 2: alizarin red staining showed the production of more calcium nodules in the 100 nM group than in the OM group on day 13. (a) Calcium nodules observed in a microscope. (b) Whole-well images of calcium nodules. (c) Relative quantification of calcium nodules performed in the whole-well images. CM: complete medium; OM: osteogenic medium; 1-100 nM E2: OM + 1-100 nM E2. Three independent experiments were performed. (Bar = 50 *μ*m. ^∗^OM group vs. 1-100 nM E2 groups, *p* < 0.05.)
