# Supplementary material for: Suppressing MicroRNA-30b by Estrogen Promotes Osteogenesis in Bone Marrow Mesenchymal Stem Cells
Source: Stem Cells Int. 2019 Apr 4;2019:7547506. doi: 10.1155/2019/7547506 (PMC6476012; doi:10.1155/2019/7547506)
Supplement: Supplementary 1 — Table 1: detailed data of the altered miRNAs. The fold change (E2/control), basal intensity values, and the normalized data of the differentially expressed miRNAs are shown. The fold change = normalized data in the E2 group/normalized data in the control group. [file 7547506.f1.doc]

**Table S1** Detail data of the altered miRNAs

| **miRNA** | **Fold change** | **Basal intensity value** | | **Normalized data** | | **miRNA** | **Fold change** | **Basal intensity value** | | **Normalized data** | |
| --- | --- | --- | --- | --- | --- | --- | --- | --- | --- | --- | --- |
|  |  | **CONTROL** | **E2** | **CONTROL** | **E2** |  |  | **CONTROL** | **E2** | **CONTROL** | **E2** |
| miR-25 | 1.83 | 646.50 | 1271.00 | 3.12 | 5.73 | miR-138-2 | 0.59 | 47.50 | 30.00 | 0.23 | 0.14 |
| let-7b | 1.77 | 1494.00 | 2841.50 | 7.22 | 12.80 | miR-214 | 0.62 | 85.50 | 56.50 | 0.41 | 0.25 |
| miR-125a | 1.50 | 781.00 | 1258.00 | 3.77 | 5.67 | miR-24 | 0.62 | 826.00 | 548.00 | 3.99 | 2.47 |
| miR-182 | 1.54 | 1147.00 | 1897.00 | 5.54 | 8.55 | miR-296 | 0.41 | 69.50 | 30.50 | 0.34 | 0.14 |
| miR-320 | 1.58 | 18.00 | 30.50 | 0.09 | 0.14 | miR-672 | 0.53 | 370.00 | 209.00 | 1.79 | 0.94 |
| miR-125b | 1.52 | 20.00 | 32.50 | 0.10 | 0.15 | miR-877 | 0.48 | 142.50 | 73.00 | 0.69 | 0.33 |
| miR-3586 | 1.51 | 43.00 | 69.50 | 0.21 | 0.31 | miR-383 | 0.59 | 65.00 | 41.00 | 0.31 | 0.18 |
| miR-673 | 2.34 | 130.50 | 328.00 | 0.63 | 1.48 | miR-490 | 0.63 | 38.00 | 25.50 | 0.18 | 0.11 |
| miR-30b | 0.22 | 771.00 | 183.00 | 3.72 | 0.82 | miR-352 | 0.65 | 568.50 | 396.00 | 2.75 | 1.78 |
| miR-322 | 0.29 | 77.50 | 24.00 | 0.37 | 0.11 | miR-28 | 0.65 | 116.50 | 81.50 | 0.56 | 0.37 |
| miR-26a | 0.49 | 607.50 | 321.00 | 2.93 | 1.45 | miR-142 | 0.67 | 187.50 | 134.00 | 0.91 | 0.60 |
| let-7i | 0.45 | 1316.50 | 628.50 | 6.36 | 2.83 | miR-195 | 0.54 | 114.00 | 66.50 | 0.55 | 0.30 |
| miR-23b | 0.62 | 1623.00 | 1086.50 | 7.84 | 4.89 | miR-34b | 0.65 | 82.00 | 57.50 | 0.40 | 0.26 |
| miR-193a | 0.58 | 676.00 | 418.50 | 3.27 | 1.89 | miR-200c | 0.60 | 369.50 | 238.00 | 1.79 | 1.07 |
| miR-141 | 0.47 | 213.00 | 107.00 | 1.03 | 0.48 |  |  |  |  |  |  |

Table S1.the fold change (E2/Control), basal intensity values and the normalized data of the differentially expressed miRNAs were shown. The fold change = normalized data in E2 group / normalized data in control group.
