# Supplementary material for: Suppressing MicroRNA-30b by Estrogen Promotes Osteogenesis in Bone Marrow Mesenchymal Stem Cells
Source: Stem Cells Int. 2019 Apr 4;2019:7547506. doi: 10.1155/2019/7547506 (PMC6476012; doi:10.1155/2019/7547506)
Supplement: Supplementary 2 — Table 2: the effect of miRNAs in previous studies. The effect of miRNAs in previous studies were presented. P: positive; N: negative; MSCs: Mesenchymal Stem Cells; BMSCs: Bone Mesenchymal Stem Cells; SMCs: Smooth muscle cells; mADSCs: mouse Adipose-Derived Stem Cells; hADSCs: human Adipose-Derived Stem Cells; hMSCs: human Mesenchymal Stem Cells; PMCO: Primary Mouse Calvaria Osteoblasts; PDLF: Periodontal Ligament Fibroblast. [file 7547506.f2.docx]

**Table S2 The effect of miRNAs in previous studies**

| miRNA | Cell | Target gene | Effect | miRNA | Cell | Target gene | Effect |
| --- | --- | --- | --- | --- | --- | --- | --- |
| miR-25 | MG63 | / | P (1) | miR-138-2 | MC3T3-E1 | EIF4BP1 | N (2) |
| let-7b | MSCs | / | P (3) | miR-214 | MSCs | FGFR1 | N (4) |
| miR-125a | BMSCs | Satb2 | N (5) | miR-24 | MC3T3 | CHI3L1 | P (6) |
| miR-182 | C3H10T1/2,MC3T3-E1 | FoxO1 | N (7) | miR-296 | / | / | / |
| miR-320 | MSCs | / | P (8) | miR-672 | / | / | / |
| miR-125b | C3H10T1/2, ST2 | Cbfβ,ErbB2 | N (5) | miR-877 | / | / | / |
| miR-3586 | / | / | / | miR-383 | / | / | / |
| miR-673 | / | / | / | miR-490 | / | / | / |
| miR-30b | MSC, SMCs | Runx2 | N (9, 10) | miR-352 | / | / | / |
| miR-322 | C2C12,MC3T3 | Tob2 | P (11) | miR-28 | / | / | / |
| miR-26a | mADSCs | Wnt5a | N (12) | miR-142 | / | / | / |
| let-7i | hMSCs | / | N (13) | miR-195 | MSCs | VEGF | N (14) |
| miR-23b | MC3T3-E1 | Smad3 | P (15) | miR-34b | PMCO | SATB2 | N (16) |
| miR-193a | hADSC | / | N (17) | miR-200c | MSCs,PDLF | IL6,IL8,  CCL-5 | P (18) |
| miR-141 | BMSCs | SVCT2 | N (19) |  |  |  |  |

Table S2. The effect of miRNAs in previous studies were presented. P: positive; N: negative; MSCs: Mesenchymal Stem Cells; BMSCs: Bone Mesenchymal Stem Cells; SMCs: Smooth muscle cells; mADSCs: mouse Adipose-Derived Stem Cells; hADSCs: human Adipose-Derived Stem Cells; hMSCs: human Mesenchymal Stem Cells; PMCO: Primary Mouse Calvaria Osteoblasts; PDLF: Periodontal Ligament Fibroblast.

1. Palmieri, A., Pezzetti, F., Spinelli, G., Arlotti, M., Avantaggiato, A., Scarano, A., Scapoli, L., Zollino, I., andCarinci, F. PerioGlas Regulates Osteoblast RNA Interfering. J Prosthodont 17, 522 2008.

2. Sun, T., Leung, F., andLu, W. miR-9-5p, miR-675-5p and miR-138-5p Damages the Strontium and LRP5-Mediated Skeletal Cell Proliferation, Differentiation, and Adhesion. International Journal of Molecular Sciences 17, 236, 2016.

3. Wei, J., Li, H., Wang, S., Li, T., Fan, J., Liang, X., Li, J., Han, Q., Zhu, L., Fan, L., andZhao, R.C. let-7 Enhances Osteogenesis and Bone Formation While Repressing Adipogenesis of Human Stromal/Mesenchymal Stem Cells by Regulating HMGA2. Stem Cells and Development 23, 1452, 2014.

4. Yang, L., Ge, D., Cao, X., Ge, Y., Chen, H., Wang, W., andZhang, H. MiR-214 Attenuates Osteogenic Differentiation of Mesenchymal Stem Cells via Targeting FGFR1. Cellular Physiology and Biochemistry 38, 809, 2016.

5. Gong, Y., Xu, F., Zhang, L., Qian, Y., Chen, J., Huang, H., andYu, Y. MicroRNA expression signature for Satb2-induced osteogenic differentiation in bone marrow stromal cells. Molecular and Cellular Biochemistry 387, 227, 2014.

6. Jin, T., Lu, Y., He, Q.X., Wang, H., Li, B.F., Zhu, L.Y., andXu, Q.Y. The Role of MicroRNA, miR-24, and Its Target CHI3L1 in Osteomyelitis Caused byStaphylococcus aureus. Journal of Cellular Biochemistry 116, 2804, 2015.

7. Kim, K.M., Park, S.J., Jung, S.-H., Kim, E.J., Jogeswar, G., Ajita, J., Rhee, Y., Kim, C.-H., andLim, S.-K. miR-182 is a negative regulator of osteoblast proliferation, differentiation, and skeletogenesis through targeting FoxO1. Journal of Bone and Mineral Research 27, 1669, 2012.

8. Wang, Y., Jiang, X.-L., Yang, S.-C., Lin, X., He, Y., Yan, C., Wu, L., Chen, G.-Q., Wang, Z.-Y., andWu, Q. MicroRNAs in the regulation of interfacial behaviors of MSCs cultured on microgrooved surface pattern. Biomaterials 32, 9207, 2011.

9. Balderman, J.A., Lee, H.Y., Mahoney, C.E., Handy, D.E., White, K., Annis, S., Lebeche, D., Hajjar, R.J., Loscalzo, J., andLeopold, J.A. Bone morphogenetic protein-2 decreases microRNA-30b and microRNA-30c to promote vascular smooth muscle cell calcification. J Am Heart Assoc 1, e003905, 2012.

10. Eguchi, T., Watanabe, K., Hara, E.S., Ono, M., Kuboki, T., Calderwood, S.K., andAhmed, S.A. OstemiR: A Novel Panel of MicroRNA Biomarkers in Osteoblastic and Osteocytic Differentiation from Mesencymal Stem Cells. PLoS One 8, e58796, 2013.

11. Gamez, B., Rodriguez-Carballo, E., Bartrons, R., Rosa, J.L., andVentura, F. MicroRNA-322 (miR-322) and Its Target Protein Tob2 Modulate Osterix (Osx) mRNA Stability. Journal of Biological Chemistry 288, 14264, 2013.

12. Li, S., Hu, C., Li, J., Liu, L., Jing, W., Tang, W., Tian, W., andLong, J. Effect of miR-26a-5p on the Wnt/Ca2+ Pathway and Osteogenic Differentiation of Mouse Adipose-Derived Mesenchymal Stem Cells. Calcified Tissue International 99, 174, 2016.

13. Vimalraj, S., andSelvamurugan, N. MicroRNAs expression and their regulatory networks during mesenchymal stem cells differentiation toward osteoblasts. International Journal of Biological Macromolecules 66, 194, 2014.

14. Almeida, M.I., Silva, A.M., Vasconcelos, D.M., Almeida, C.R., Caires, H., Pinto, M.T., Calin, G.A., Santos, S.G., andBarbosa, M.A. miR-195 in human primary mesenchymal stromal/stem cells regulates proliferation, osteogenesis and paracrine effect on angiogenesis. Oncotarget 7, 7, 2016.

15. Liu, H., Hao, W., Wang, X., andSu, H. miR-23b targets Smad 3 and ameliorates the LPS-inhibited osteogenic differentiation in preosteoblast MC3T3-E1 cells. J Toxicol Sci 41, 185, 2016.

16. Wei, J., Shi, Y., Zheng, L., Zhou, B., Inose, H., Wang, J., Guo, X.E., Grosschedl, R., andKarsenty, G. miR-34s inhibit osteoblast proliferation and differentiation in the mouse by targeting SATB2. The Journal of Cell Biology 197, 509, 2012.

17. Zhang, Z.J., Zhang, H., Kang, Y., Sheng, P.Y., Ma, Y.C., Yang, Z.B., Zhang, Z.Q., Fu, M., He, A.S., andLiao, W.M. miRNA expression profile during osteogenic differentiation of human adipose-derived stem cells. J Cell Biochem 113, 888, 2012.

18. Hong, L., Sharp, T., Khorsand, B., Fischer, C., Eliason, S., Salem, A., Akkouch, A., Brogden, K., andAmendt, B.A. MicroRNA-200c Represses IL-6, IL-8, and CCL-5 Expression and Enhances Osteogenic Differentiation. PLOS ONE 11, e0160915, 2016.

19. Sangani, R., Periyasamy-Thandavan, S., Kolhe, R., Bhattacharyya, M.H., Chutkan, N., Hunter, M., Isales, C., Hamrick, M., Hill, W.D., andFulzele, S. MicroRNAs-141 and 200a regulate the SVCT2 transporter in bone marrow stromal cells. Molecular and Cellular Endocrinology 410, 19, 2015.
