# Supplementary figures and images for: Suppressing MicroRNA-30b by Estrogen Promotes Osteogenesis in Bone Marrow Mesenchymal Stem Cells
Source: Stem Cells Int. 2019 Apr 4;2019:7547506. doi: 10.1155/2019/7547506 (PMC6476012; doi:10.1155/2019/7547506)

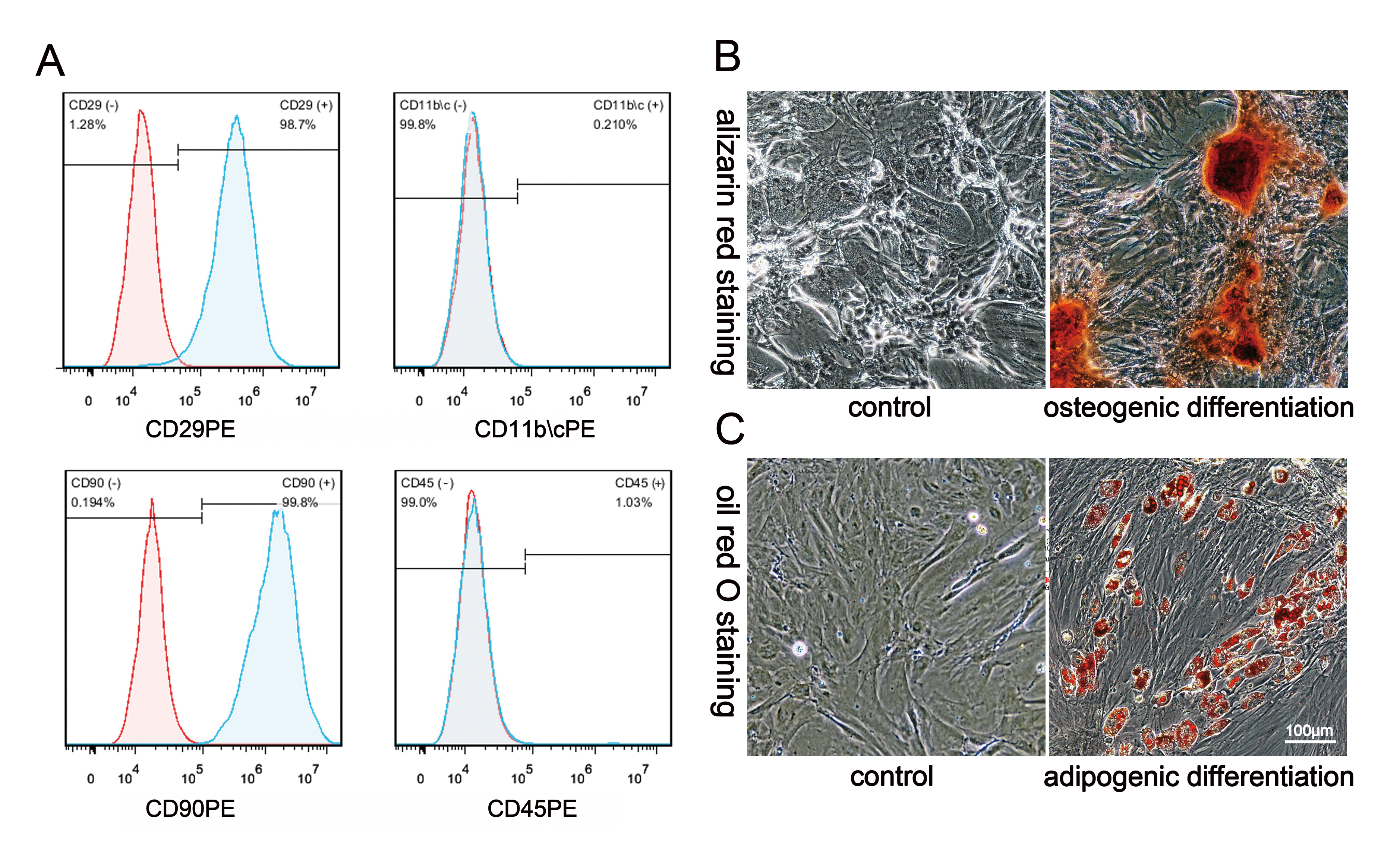

Supplement: Supplementary 3 — Figure 1: identification of rBMSCs. (a) Flow cytometry assay shows the surface markers of rBMSCs. Isotype controls are presented as red plots, and the specific cell surface markers are presented as blue plots. The isolated rBMSCs were positive for CD29 and CD90, both of which are the markers of rBMSCs, but were negative for myelogenous makers CD11b/c and CD45. (b, c) The multidirectional differentiation abilities of rBMSCs. (b) The alizarin red staining showed that rBMSCs cultured in the osteogenic differentiation medium displayed significant calcium deposits compared with the control group, and (c) the oil red O staining showed that rBMSCs cultured in adipogenic differentiation medium could accumulate lipid droplets compared with the control group. Three independent experiments were performed. (Bar = 100 μm.) [file 7547506.f3.tif]

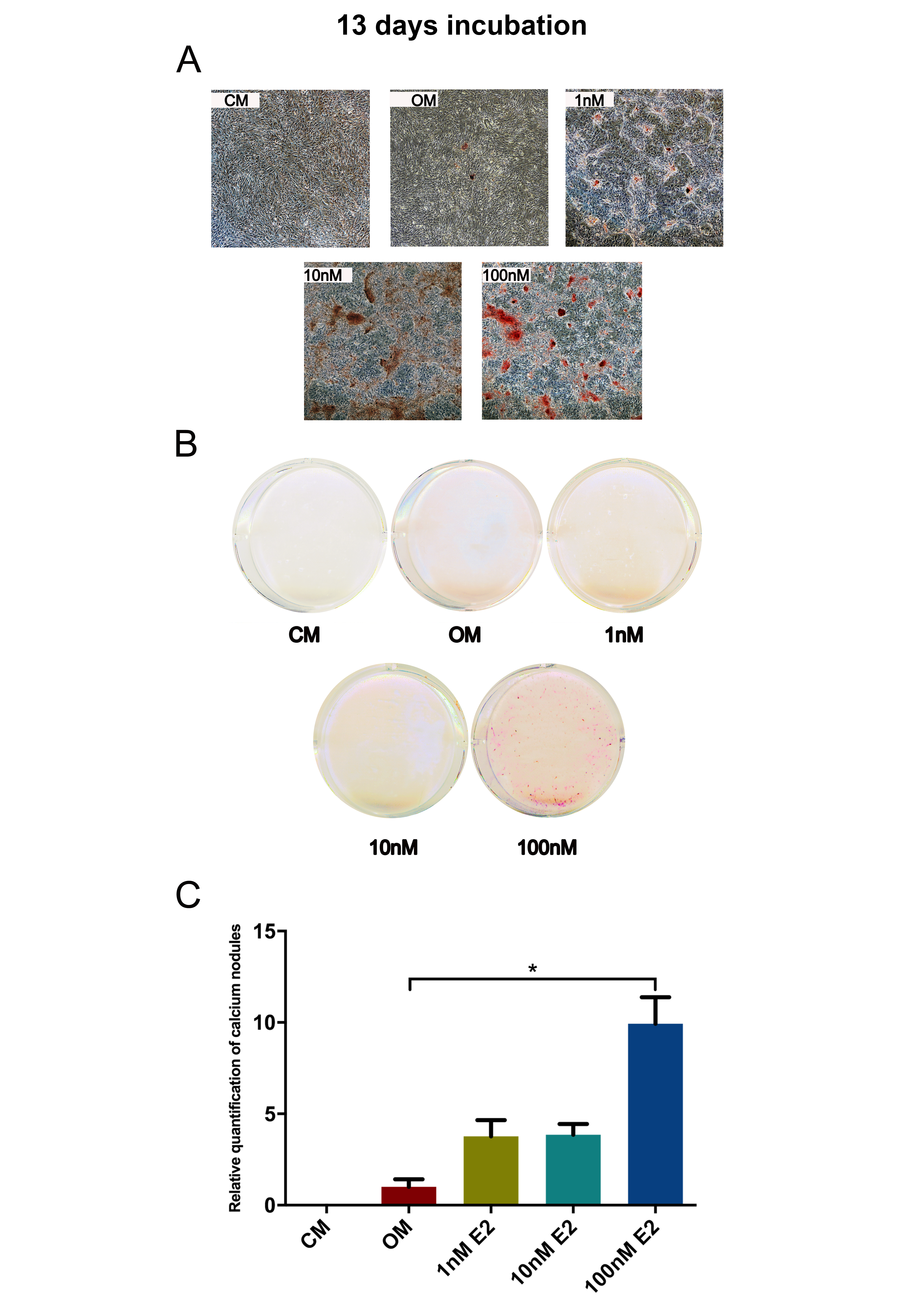

Supplement: Supplementary 4 — Figure 2: alizarin red staining showed the production of more calcium nodules in the 100 nM group than in the OM group on day 13. (a) Calcium nodules observed in a microscope. (b) Whole-well images of calcium nodules. (c) Relative quantification of calcium nodules performed in the whole-well images. CM: complete medium; OM: osteogenic medium; 1-100 nM E2: OM + 1-100 nM E2. Three independent experiments were performed. (Bar = 50 μm. ∗OM group vs. 1-100 nM E2 groups, p < 0.05.) [file 7547506.f4.tif]
